# Implementation and sustainability of safe consumption sites: a qualitative systematic review and thematic synthesis

**DOI:** 10.1186/s12954-022-00655-z

**Published:** 2022-07-05

**Authors:** Grace H. Yoon, Timothy W. Levengood, Melissa J. Davoust, Shannon N. Ogden, Alex H. Kral, Sean R. Cahill, Angela R. Bazzi

**Affiliations:** 1 Department of Health, Law, Policy and Management, Boston University School of Public Health, 715 Albany St, Boston, MA 02118 USA; 2 RTI International, 2150 Shattuck Avenue. Suite 800., Berkeley, CA 94704 USA; 3 Fenway Institute and Department of Health, Law, Policy and Management, Boston University School of Public Health, 715 Albany St, Boston, MA 02118 USA; 4 Herbert Wertheim School of Public Health and Human Longevity Science, University of California, 9500 Gilman Dr. La Jolla, San Diego, CA 92093 USA; 5 Department of Community Health Sciences, Boston University School of Public Health, 715 Albany St, Boston, MA 02118 USA

**Keywords:** Implementation science, Harm reduction, Safe consumption sites, Supervised consumption sites, Drug consumption rooms, Qualitative, People who use drugs

## Abstract

**Background:**

Safe consumption sites (SCSs) serve diverse populations of people who use drugs (PWUD) and public health objectives. SCS implementation began in the 1980s, and today, there are at least 200 known SCSs operating in over twelve countries. While a growing literature supports their effectiveness as a harm reduction strategy, there is limited information on contextual factors that may support or hinder SCS implementation and sustainability*.* We aimed to fill this gap in knowledge by reviewing existing qualitative studies on SCSs.

**Methods:**

We conducted a systematic review and thematic synthesis of qualitative studies. We identified all peer-reviewed, English-language qualitative studies on SCSs containing original data in *PubMed, Web of Science, Google Scholar,* and *Science Direct* as of September 23, 2019. Two authors independently screened, abstracted, and coded content relating to SCS implementation and sustainment aligned with the Exploration, Preparation, Implementation, Sustainment (EPIS) implementation science framework.

**Results:**

After removing duplicates, we identified 765 unique records, of which ten qualitative studies met inclusion criteria for our synthesis. Across these ten studies, 236 total interviews were conducted. Overall, studies described how SCSs can (1) keep drug use out of public view while fostering a sense of inclusion for participants, (2) support sustainment by enhancing external communities’ acceptability of SCSs, and (3) encourage PWUD utilization. Most studies also described how involving PWUD and peer workers (i.e., those with lived experience) in SCS operation supported implementation and sustainability.

**Discussion:**

Our thematic synthesis of qualitative literature identified engagement of PWUD and additional factors that appear to support SCS planning and operations and are critical to implementation success. However, the existing qualitative literature largely lacked perspectives of SCS staff and other community members who might be able to provide additional insight into factors influencing the implementation and sustainability of this promising public health intervention.

## Background

Globally, there are an estimated 270 million people who use drugs (PWUD) [1]. Safe consumption sites (SCSs)—also called drug consumption rooms, supervised consumption sites, or supervised injection facilities—allow PWUD to use pre-obtained substances under the supervision of trained health workers, while reducing public visibility and unnecessary police intervention [2]. First implemented in the 1980s in Switzerland, there are likely more than 200 SCSs operating in at least twelve countries today [3]. Services provided in SCSs vary, but often include the provision of sterile drug consumption equipment, disposal methods, and drug checking services. They may also include counseling on safe drug use, infectious disease testing, and referrals to healthcare, substance use disorder treatment, and other social services [4].

Several systematic reviews summarize the effectiveness and safety of SCSs. One early review by Kerr and colleagues (2007) examined the impact of SCSs on HIV prevention outcomes, finding that SCSs helped reduce syringe sharing and unsafe syringe disposal [2]. A seminal review by Potier et al. (2014) concluded that SCS can effectively promote safety among PWUD without encouraging drug use or drug distribution within surrounding communities [5]. Kennedy, Karamouzian and Kerr (2017) found that SCSs also have positive impacts for communities in which they are implemented by connecting PWUD with health and social services and reducing public order and street safety concerns [6]. Additional reviews by Caulkins (2019) and Pardo (2018) found no evidence of adverse events within the sites or in the wider community due to SCS presence [7, 8]. Levengood and colleagues’ (2021) systematic review found that SCSs reduced overdose mortality and morbidity while having no negative impact on public safety [9, 10].

Beyond evidence of SCS effectiveness for health and safety outcomes, recent reviews have investigated pre-implementation considerations for the establishment of SCSs, including acceptability and feasibility. In 2019, Lange and Bach-Mortensen’s systematic review pointed out differing perceptions of benefits and concerns among different SCS stakeholders (i.e., police compared to PWUD) [11]. In 2021, Xavier and colleagues’ review of SCS feasibility studies concluded that, prior to implementation, SCSs should have minimal eligibility criteria and institutional restrictions in order to maximize benefits to PWUD and broader communities [12]. A qualitative synthesis of studies in five U.S. jurisdictions highlighted the importance of early community engagement, organizing people with lived experience, securing political champions, and building coalitions to gather political momentum [13]. An article recently published in January 2022 provided a scoping review of SCS design preferences, such as location, hours, and wait times, as reported by PWUD [14]. Contrary to the traditional SCS role of promoting safe injection, SCSs in the recent era have increasingly embraced non-injection forms of drug use, such as inhalation [15]. However, to our knowledge, no papers have systematically reviewed existing qualitative studies examining factors that hinder or support the actual implementation or sustainability of these evidence-based public health interventions.

## Methods

### Systematic review methods

To inform public health policy and practice, we conducted a systematic review and thematic synthesis of qualitative studies guided by the Exploration, Preparation, Implementation, and Sustainment (EPIS) framework [16]. EPIS is a multilevel, four-phase approach to the implementation of evidence-based practices. Earlier reviews have established the evidence base of SCSs for public health and safety outcomes and explored the earlier exploration and preparation phases, which involve considering sociopolitical contexts, initial funding sources, staff recruitment and training, and leadership [17]. We build on existing evidence by identifying and synthesizing rich contextual data on SCS implementation and sustainment (Table 1) [17].

**Table 1:** Operational definitions of EPIS parent and subcodes (Moullin et al., 2019)

Term	Operational definition
Implementation	Active implementation processes at a systems-level, including factors related to funding, legality, workforce productivity, and user feedback
Sustainment	Factors that support continuous EBP delivery–with adaptations as necessary–to achieve lasting public health impact, including factors related to long-term financial support and/or self-sufficiency
Outer context	The environment external to the organization; service and policy environments and characteristics; inter-organizational relationships between governments, funders, managed care organizations, professional societies, advocacy groups
Inner context	Characteristics within an organization; leadership (high vs middle management), staffing (paid clinicians vs peer volunteers), facility-specific practices, individual adopters/ practitioners
Bridging factors	The interconnectedness and relationships between outer and inner context entities influence the implementation process as outer and inner processes influence each other in a reciprocal nature
Innovation	The evidence-based practice or intervention itself, or novel parts of it; fit of the intervention with the system and target population (outer system) and the organization itself and its providers (inner context); any adaptations necessary to maximize the intervention’s fit. After the initial opening of the SCS, innovation factors may be implemented for improved access and operations and help the SCS be more sustainable for longer and wider use

Our search for relevant articles followed Preferred Reporting Items for Systematic Reviews and Meta-Analyses (PRISMA) guidelines [18]. Our search strategy was based on an earlier systematic review focused on quantitative and qualitative studies of safe injection facilities by Potier and colleagues, as previously described and detailed in “Appendix 1” [5]. We took studies included in Potier’s original review, added more recent studies found by updating Potier’s search period, applied our specific inclusion criteria, and analyzed the resulting included studies using the EPIS framework.

To build on Potier’s review, we expanded the focus from injection to other forms of drug consumption (e.g., inhalation, snorting, smoking) and extended their original search period (from database inception to January 26, 2014) through September 23, 2019. This search (“Appendix 1”) identified 22 quantitative effectiveness studies, reviewed elsewhere [9], and a large body of descriptive qualitative literature. The qualitative studies identified through this initial search provided rich contextual data not captured in the existing quantitative reviews; therefore, we deemed this body of qualitative literature worthy of a separate systematic review to identify common contexts and processes relevant to SCS implementation and sustainment. This qualitative review and thematic synthesis also involved screening the references included papers to identify additional relevant studies.

We identified and eliminated duplicate records at the pre-screening stage. We included English language, peer-reviewed papers reporting original data from qualitative studies of already existing, operational SCS, which we defined as established facilities where PWUD could use substances via any route of administration (e.g., injection, inhalation, smoking). We excluded articles not relevant to specific, operational SCS or the EPIS model’s Implementation or Sustainment phases based on collective judgment of the analytic team (e.g., mathematical modeling studies of potential impacts of hypothetical facilities) [19]. Studies with the same authors, settings, and samples were pooled and considered as one study. Four members of the analytic team (GY, TL, SO, MD) were involved in title and abstract screening, retrieval and review of full-text articles, and quality assessment using the Critical Assessment Skills Programme (CASP) checklist [20]. The CASP assessment involved a qualitative review of the study’s aims, appropriateness of methodology and design, ethical considerations, analyses, and overall value of the study. Two members of the analytic team independently reviewed and reconciled their CASP screening and quality assessment findings.

### Thematic synthesis methods

We developed a codebook directly from the established EPIS framework with Implementation and Sustainment parent codes that each had four child codes for (1) outer context, (2) inner context, (3) bridging factors, and (4) innovation factors [17, 21]. No additional child codes were created during the coding or analysis processes. Two members of the analytic team (combinations among GY, TL, SO, and MD) independently reviewed and coded qualitative data from all included articles. The team met weekly to review consistency in coding and reconcile any differences in coding. We then organized all coded data into a table aligned with the EPIS framework. Finally, we conducted axial coding of the organized data to identify generalizable themes across the more granular codes described above in order to identify potential best practices for SCS implementation and sustainment [22].

## Results

**Fig. 1:**
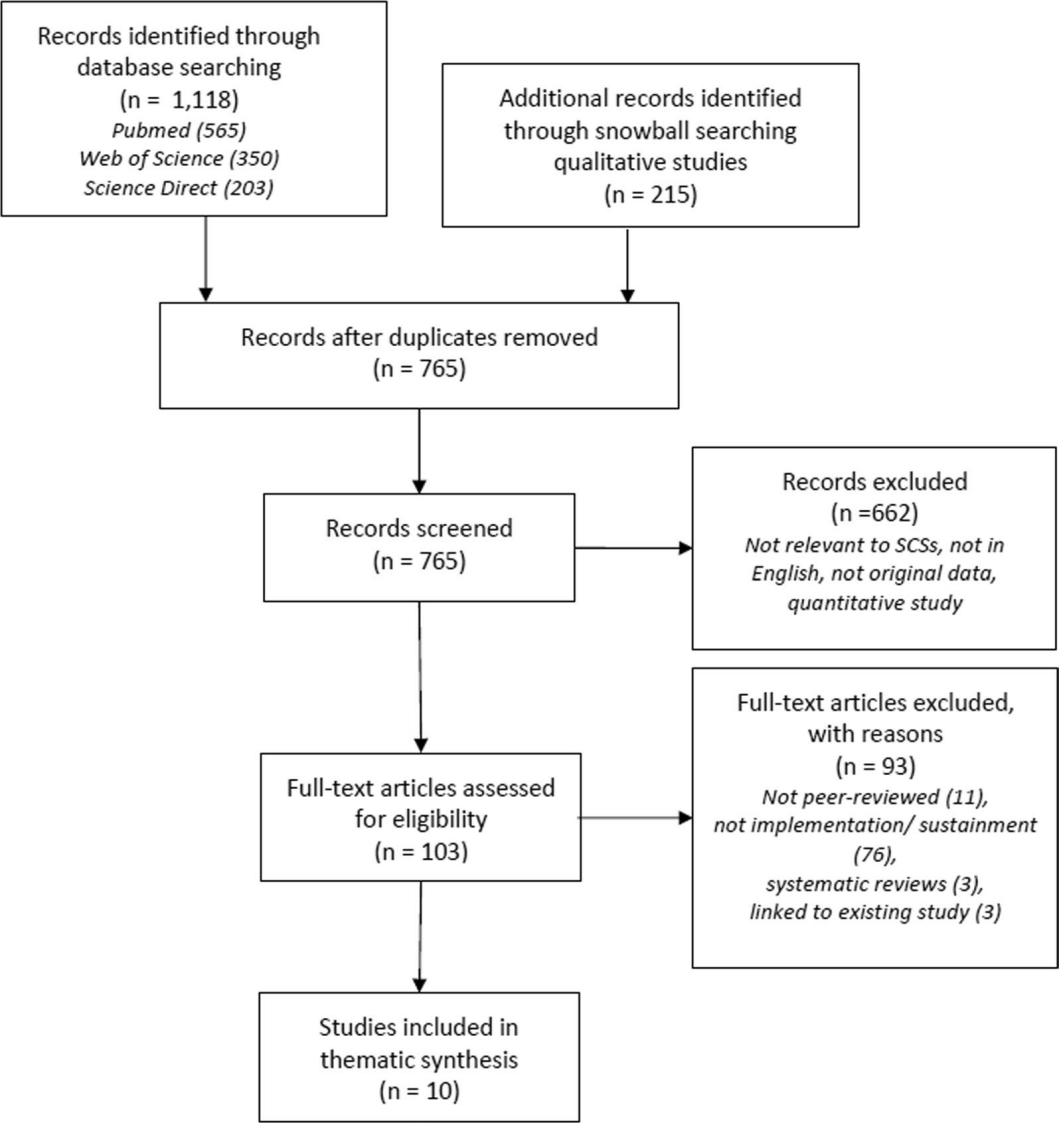
This figure follows the recommended PRISMA diagram for systematic reviews. The top left box notes the studies found using the base search strategy that returned both quantitative and qualitative studies. The top right box notes additional qualitative studies that were snowballed from the reference list of studies selected for a separate quantitative review exclusive to safe injection sites. PRISMA diagram

From 765 unique records, 10 qualitative studies representing nine SCSs in five countries met inclusion criteria (Fig. 1). All ten studies (Table 2) used qualitative methods resulting in a pooled total of 236 participant semi-structured interviews. Two of these studies also utilized participant observation methods (approximately 50 h of participant observation in Canada [23] and 12 months of participatory ethnographic fieldwork in Germany) [24]. One study from Italy solely utilized weekly diaries of participant observation over a period of ten years since the SCS’s inception [25]. Overall, 22% of participants were staff or peer workers, and the rest were SCS participants (i.e., PWUD who accessed the SCSs to utilize the spaces). Aside from a cluster of early manuscripts (n = 4) published between 2006 and 2009 originating from one cohort study (SEOSI) in Vancouver [26–29], the other nine studies were published between 2014 and 2019 [23–25, 30–35]. Six studies were from Canada [23, 26–32, 35], and one study each was from Denmark [33], the United States [34], Italy [25], and Germany [24].

**Table 2:** List of included studies and summary of findings

Author	Year	Country	CASPa score (x/10)	Implementation				Sustainment			
				Outer	Bridging	Innovation	Inner	Outer	Bridging	Innovation	Inner
Jozhaghi	2016	Canada	8			×	×	×			
McNeil	2015	Canada	8	×	×		×	×			
Kappel^a^	2016	Denmark	7		×	×	×				×
McNeil	2014	Canada	9		×	×	×				
Davidson	2018	U.S	7	×	×	×	×	×	×	×	×
McNeil	2014	Canada	8		×	×	×	×			
Kennedy	2019	Canada	7	×	×	×	×				×
Bergamo	2019	Italy	5	×		×	×	×			
Duncan	2017	Germany	7	×		×	×				
SEOSI^b^	2006–2009	Canada	7–8	×	×	×	×	×			

This study represents data from five Danish sites

^**a**^Critical Assessment Skills Programme

^b^This is a combination of four studies published by members of Scientific Evaluation of Supervised Injecting, or SEOSI (Fast et al. 2008, Kerr et al. 2007, Small et al. 2008, Small et al. 2009)

Our CASP quality assessment results were generally positive, with some common limitations across studies, including: failure to discuss relationships between researchers and participants (i.e., reflexivity), non-systematic recruitment strategies (e.g., depending entirely on investigators’ rapport with specific participants), and limited engagement of participants in data analysis or interpretation [24]. Our thematic synthesis identified key aspects of SCS implementation and sustainment pertaining to outer and inner contexts, along with bridging and innovation factors, as detailed below and summarized in Table 3.

**Table 3:** Implementation and Sustainability findings according to EPIS components

Phase	Outer context	Inner context	Bridging factors	Innovation factors
Implementation	1. Community buy-in on the need for improved harm reduction infrastructure 2. Framing SCSs as a tool to reduce the visibility of drug use in surrounding communities (a shared goal of participants and community members)	1. Workforce a. Encouraging mutual respect between SCS clients and workers b. Addressing power imbalances 2. Participant experience a. Fostering sense of community b. Designating a time and space for drug use c. Reducing fear of adverse consequences	1. Peer workers a. Community volunteers b. Social workers 2. Relaxed rules and regulations within SCSs 3. Establishing connections with outside agencies	1. Building social connections among participants 2. Modifying physical spaces to increase participant comfort and socialization 3. Providing safety and harm reduction counseling 4. Offering services with the lowest possible barriers to access
Sustainment	1. Maintaining community relationships 2. Providing unique resources to PWUD 3. Framing SCSs as a cost-saving intervention	1. Specific pathways for increasing social capital for PWUD 2. Adequate support for peer-workers 3. Finding balance between the desires of mainstream oversite and the needs of the most-marginalized participants	1. Discreet community outreach efforts 2. Building trust and acceptance with participants, treatment partners, and broader community	1. Maximizing accessibility a. fewer regulations b. longer hours 2. Training participants to reduce drug harms beyond injection (i.e., inhalation) 3. Providing additional private consumption spaces (e.g., for accessing certain injection sites such as the groin), 4. Co-location of health and social services 5. Availability of drug testing services

### SCS implementation

#### Outer context

Outer contextual factors—defined as characteristics of service or policy environments outside of organizations—that facilitated SCS implementation included: (1) community buy-in on the need for improved harm reduction infrastructure, and (2) framing SCSs as a tool to reduce the visibility of drug use in surrounding communities, a shared goal of participants and community members. In the successful implementation examples described, key external players identified in studies included supportive policymakers who ultimately decided which types of SCSs would be allowed in their jurisdictions, and clinical providers with positive attitudes toward PWUD within and outside of SCSs.

Six studies described outer contextual factors supporting SCS implementation; each highlighted the role of local funders and physical environments in which PWUD lived and used drugs [23, 24, 26–29, 34, 35]. One Canadian SCS was primarily funded by the local health department, but only after PWUD reported that the old, informal SCS space was limited and disconnected from other social services [23]. In that case, harm reduction advocates persuaded an external entity to provide funding, enabling the expansion of resources and establishment of a larger, improved SCS. Another study from an unsanctioned U.S. SCS cited implicit, informal support from local police and community members who defended the SCS if authorities accused the site or its clients of illicit behaviors [34]. At another Canadian site, local community members were supportive because they perceived the SCS to decrease the harms of unsafe and rushed drug use in their community [26–29].

Studies also described how SCSs played a role in reducing the visibility of drug use in surrounding communities, which may have benefited both PWUD and other community members. SCS participants described that the privacy provided by SCSs increased their comfort and feelings of dignity. For broader communities, studies described how SCSs reduced the visibility and “nuisance” of public drug use (e.g., exposure to witnessing drug use).

#### Inner context

Inner contextual factors, defined as characteristics of the culture, structures, and practice within organizations, that impacted SCS implementation included: (1) the workforce, including the importance of mutual respect between SCS participants and workers (i.e., addressing power imbalances), and (2) the participant experience, including fostering a sense of community, designating a space and time for drug use, and reducing fear of adverse consequences. Most studies described the need to adequately support peer workers (i.e., individuals with lived experience with drug use), and challenges regarding internalized stigma among PWUD toward their own drug use, which could negatively influence their SCS experiences [24–28, 30–34]. Studies from Canada described how peer workers foster social cohesion and security within SCSs [30–32, 35]. Peer workers also helped to reduce internalized stigma among PWUD, countering feelings of exclusion PWUD commonly experienced in clinical and social service settings. In one German SCS, PWUD “felt respite from the stigma of ‘junkie’ identities,” and described being able to more fully experience the psychological and physiological effects of drugs [24]. Experiences with safer drug use also helped PWUD recognize and avoid unsafe situations during street drug use. In an unsanctioned, PWUD-run SCS in Italy, participants felt empowered when helping peers, particularly when they were able to intervene in harmful situations, like reversing overdoses [25]. In this context, PWUD would even visit the SCS without using drugs. Similarly, a study representing five Danish SCSs found that, aside from increasing safety, SCSs promoted social cohesion by providing a space where PWUD could gather and share information about employment, housing, and other resources [33].

#### Bridging factors

Seven studies discussed bridging factors that connected outer and inner contexts to support SCS implementation. These included: (1) peer workers (and community volunteers and social workers), (2) relaxed rules and regulations within SCSs, and 3) establishing connections with outside agencies (e.g., by connecting PWUD to health and social services) [2, 23, 26, 28, 31–34, 36]. First, peer workers supported SCS implementation by providing nuanced expertise in reducing drug-related harms and relaying information on social resources that may not be accessible via traditional clinical or social services [35]. In Vancouver, volunteer peer workers brought PWUD in from the streets, reducing community disruption and violence between police and PWUD [35]. Second, several studies noted that loosened regulations were more appealing to PWUD, while SCSs with more rules (e.g., against smoking or injection assistance) deterred higher-risk individuals who could have most benefitted from SCSs [12]. For example, an unsanctioned Canadian SCS that relaxed rules prohibiting assisted drug administration experienced improved engagement from disadvantaged groups of PWUD including those living with disabilities, individuals injecting in the groin or neck, and youth who could not meet age requirements at a sanctioned site [23]. Finally, clinical and professional SCS staff linked PWUD to health and social services, including infectious disease testing, which further connected SCSs (and their clients) to external agencies [33].

#### Innovation factors

All ten studies described innovations supporting SCS implementation, including: (1) building social connections among participants, (2) modifying physical spaces to increase PWUD comfort (e.g., café or place to relax), (3) providing safety and harm reduction counseling, and 4) offering services with the lowest possible barriers to access. All studies described the involvement of peer workers as vital to establishing and improving upon these innovations [6, 23–34]. For example, at a sanctioned Canadian site, peers provided detailed harm reduction education [30]. At another unsanctioned Canadian site, peers provided equipment to clients upon entry, counseling prior to drug administration, and oversight of person and time limits within physical spaces [23]. Peers in the unsanctioned U.S. site provided education regarding obtaining and using clean equipment and naloxone [34]. Additional innovations that were implemented included vein detection technology for safe injection (Denmark), dual-level entrances (e.g., one accessible anytime for safe equipment disposal, another open during SCS operating hours for full services; Italy), and co-location of a non-clinical “café” social space where SCS staff and clients could interact and access low-cost refreshments (Germany) [24, 25, 33].

### SCS sustainment

#### Outer context

Five studies included outer contextual factors supporting SCS sustainment that involved (1) continued community support by reducing visibility of substance use in the community, (2) providing resources based on PWUD-identified needs, and (3) presenting SCS as an overall cost-saving intervention by preventing drug-related health and public order issues [25–30, 32, 34]. One study described how a sanctioned SCS in Canada benefitted from long-term public funding generated by community activism following the forced closure of an unsanctioned site. This site was also the focus of many peer-reviewed academic studies that reported on its positive clinical and social effects, which further validated the SCS’s presence and may have supported sustainment [6]. A study of an unsanctioned U.S. site concluded that the underground nature of their site decreased “not in my backyard” sentiments in the surrounding community, ultimately supporting the likelihood of sustainment [34]. In addition, a study of an Italian SCS concluded that authorities’ gradually increasing recognition of the public health benefits and lack of complaints from community members supported sustainment [25].

#### Inner context

Three studies described inner contextual factors related to SCS sustainment, including (1) pathways for increasing social capital for PWUD, (2) support for peer workers, and (3) fears of barriers to entry as SCS became more mainstream and imposed more regulations upon clients [33–35]. In Denmark, participants noted that organizational goals (e.g., entering drug treatment, reintegrating with society) could support sustainment [33]. Maintaining a focus on their harm reduction mission provided a basis upon which new adaptations could be made, such as the decisions to provide “humanizing” interactions (rather than framing services as clinical supervision) and maintain a low-threshold facility to reduce barriers to access and connect PWUD to informational and preventative resources in the community. The U.S. study found that the unsanctioned nature of the site provided some flexibility due to its invisibility from law enforcement, and participants expressed concerns about increased legal consequences and barriers to SCS use if the site became sanctioned and subjected to increased oversight [34]. In Canada, researchers argued that SCS sustainment would depend on the treatment and involvement of peer workers, calling for their services to be met with proper compensation, training, and physical and mental health supports [35].

#### Bridging and innovation factors

The study of the unsanctioned U.S. site described bridging and innovation factors, including discreet community outreach efforts to ensure equitable access to the site and referrals to health and social services, that supported SCS sustainability by raising acceptability within local medical and residential communities [34]. Potential innovation factors generated by SCS participants at the U.S. site included improved accessibility (e.g., via fewer regulations and longer hours), relevant training on reducing non-injection drug-related harms, additional private spaces (e.g., for accessing certain injection sites such as the groin), co-location of health and social services, and availability of drug testing services. All participant recommendations responded to current PWUD needs in the community and, if implemented, would encourage continued use and access of SCS services. A Canadian SCS provided supportive care services with residential beds; participants at the site identified that the site’s designation as a healthcare facility could contribute to its sustainment [32].

## Discussion

As evidence on the effectiveness of SCSs for reducing overdose deaths and drug-related harm has become clearer, local policymakers and public health planners have become increasing interested in implementing SCSs [9, 37–39]. Our systematic review and thematic synthesis of qualitative studies from diverse settings identified some contextual factors that may influence SCS implementation and sustainment. This synthesis of rich contextual data suggests the need for additional research into specific programmatic, policy, and advocacy efforts that could support the scale-up of this promising but underutilized public health intervention, as discussed below.

First, our findings underscore how SCS implementation efforts may meet “not in my backyard” (i.e., “NIMBY”) sentiments within local communities [40]. This potential challenge to SCS implementation was best exemplified by the unsanctioned U.S. site that engaged local law enforcement support [34], suggesting that external buy-in prior to SCS implementation could be useful, particularly in neighborhoods where community members feel unsafe with high prevalence of visible street drug use. When implemented, SCSs can achieve dual goals, reducing public visibility and consequences of drug use while fostering a sense of inclusion, and socialization among PWUD. Increased quantitative and qualitative (i.e., mixed methods) evaluations of operational SCSs could provide more comprehensive evidence on specific geographic and demographic differences in implementation, enabling the adoption of SCSs for different PWUD communities.

Next, we found that SCS sustainment was supported by the fostering of environments that ensured continued acceptability and utilization within the PWUD community, increased safety, and support among local community members. The provision of various health and social service referrals, particularly to substance use disorder treatment services, could help promote positive perceptions of SCSs within local communities. An unsurprising facilitator of SCS sustainment identified in the literature we reviewed was continued legal and political support, often bolstered by local data regarding law enforcement and community members’ positive perceptions of SCSs. Future research should investigate and identify key influencing factors in financing, policing, and surveilling of SCSs. External perspectives on SCS implementation that warrant additional research include funding agencies, law enforcement, and legal experts given the vast differences in drug policies and their implementation across contexts. There are existing examinations of influential institutions and external decision-makers in other countries where SCSs have been debated and implemented, like Finland [41] and Belgium [42]. In a time of frequent policy debate regarding harm reduction in the United States [43–45], future research should consider the role of local laws, their enforcement, and broader political sentiments surrounding SCS implementation.

While lessons regarding SCS implementation and sustainment drawn from studies in Canada and Europe might provide some helpful insights for other legal and political contexts, additional research in diverse international settings is clearly needed to improve the generalizability and transferability of this literature. Diverse socio-political contexts may vary in their tolerance of harm reduction approaches and endorsement of moralizing narratives surrounding substance use [46]. There is evidence that these moralistic views are difficult to change, even with robust scientific evidence to contradict such beliefs [47]. Recent evidence suggests that policymakers are more encouraged to pursue interventions such as a SCS in their local communities in the wake of new evidence of success from other harm reduction interventions that have been evaluated in their jurisdictions [13].

Importantly, the involvement of PWUD and peer workers (i.e., those with lived experience) in SCS implementation and sustainment emerged as an important cross-cutting theme in our review of qualitative evidence. According to the literature we synthesized, peer workers may be overlooked in efforts to implement and sustain SCSs, despite abundant evidence that they bring critical expertise and effort into these services. To perform their critical functions, peer workers require adequate compensation and recognition, including in the form of formal employment and workplace occupational supports for physical and mental health. Additional research on the optimal engagement of peer workers within SCSs and harm reduction programming, particularly as it relates to sustainment, is needed.

Several limitations of our study warrant consideration. First, consistent with the broader implementation science literature grounded in the EPIS framework [48], we identified more detailed evidence on implementation than sustainment. Less evidence was available on outer contextual, innovation, or bridging factors, particularly related to sustainment. These studies were also represented in Potier’s original review, but we believe the current study frames these studies in a novel way using the EPIS framework. Second, we excluded non-English studies and gray literature, and most published SCS research originated from Canada and Europe. Government reports, particularly from Europe, often describe SCS implementation in greater detail than what is represented in the academic literature we reviewed; these types of reports, which may include data based on surveys with SCS participants [49] and managers [50], could contain relevant information but were out of the scope of this review. Notably, reports from community members (e.g., in Australia [51–53]) have highlighted the importance of participant input into facility regulations, mirroring some of the sustainment-related findings of our review. Given the rather limited range of contexts in which the studies included in our were conducted, additional review of non-English studies and gray literature (particularly including surveys of SCS participants, managers, staff, and community members) could help contextualize or expand upon our findings, ultimately improving the transferability of this work. Third, given the focus on safe injection sites in our initial search strategy, we may have missed qualitative studies related to SCS implementation for other forms of drug administration; however, our additional screening process through references of initially included studies for relevant work helped mitigate this limitation. Finally, the final updated search was completed in September 2019, leaving a considerable gap to publication and missing the critical period when the COVID-19 pandemic likely impacted SCS operations. Additional research on this more recent period is needed to understand factors influencing SCS implementation and sustainability during a large-scale public health crisis. Furthermore, while relationships with police and law enforcement emerged in several studies in our review, the broader literature on SCS and other harm reduction interventions highlights it with greater prominence that what appeared in our sample of studies; additional research is needed to systematically investigate the impact of law enforcement relationships on the implementation and sustainment of SCS and other harm reduction interventions.

Notwithstanding these limitations, our systematic review and thematic synthesis of qualitative studies identifies some of the key factors that have supported and challenged SCS implementation and sustainment around the world. We identified that engaging PWUD in SCS design and implementation can contribute to the sense of community and mutual respect found in successful SCSs. In addition, encouraging social cohesion among clients and connecting them to outside agencies supports SCS implementation and sustainment. Although evidence was limited regarding SCS sustainability, contributing factors included visibly reducing drug use and improving safety for local communities while increasing the dignity of PWUD. Finally, community outreach efforts to ensure equitable access to SCS facilities represented an important bridging and innovation factor supporting sustainability.

As more healthcare professionals, community advocates, and policymakers consider SCSs as a strategy to reduce drug-related health harms, high-quality research on the implementation and sustainability SCSs in different localities is critical. By identifying key factors in the implementation process, improved SCS implementation and sustainment can be realized in communities where these services may be of great benefit.

## Appendix 1: Search strategy for each database

**Table:**

Database	Dates	Strategy
PubMed	1/1/2014–9/23/2019	((“SUPERVISED” [All Fields] OR “SAFER” [All Fields]) AND (“INJECTION” [All Fields] OR “INJECTING” [All Fields] OR “SHOOTING” [All Fields] OR “CONSUMPTION” [All Fields]) AND (“FACILITY” [All Fields] OR “FACILITIES” [All Fields] OR “ROOM” [All Fields] OR “GALLERY” [All Fields] OR “CENTRE” [All Fields] OR “CENTER” [All Fields] OR “SITE” [All Fields])) AND (2014:2019 [pdat])^a^
Web of Science	1/1/2014–9/23/2019	TS = ((“SUPERVISED” OR “SAFER”) AND (“INJECTION” OR “INJECTING” OR “SHOOTING” OR “CONSUMPTION”) AND (“FACILITY” OR “FACILITIES” OR “ROOM” OR “GALLERY” OR “CENTRE” OR “CENTER” OR “SITE”)) Indexes = SCI-EXPANDED, SSCI, A&HCI, CPCI-S, CPCI-SSH, BKCI-S, BKCI-SSH, ESCI Timespan = 2014-2019^a^
Science Direct	1/1/2014–9/23/2019	Year: 2014-2019^a^ Title, abstract, keywords: (“SUPERVISED” OR “SAFER”) AND (“INJECTION” OR “INJECTING” OR “CONSUMPTION”) AND (“FACILITY” OR “FACILITIES” OR "SITE”) (note: max seven Boolean operators) (note: Boolean operator limit, had to reduce terms) Article types: Research articles Refine by subject areas: Medicine and Dentistry

^a^Original search only included 2019 studies up to search date 9/23/2019. Coarser full-year database filters may thus result in search yields with slightly larger number of studies (includes to end of 2019).
